# Role of Transforming Growth Factor-*β*1 and Smads Signaling Pathway in Intrauterine Adhesion

**DOI:** 10.1155/2016/4158287

**Published:** 2016-02-21

**Authors:** Umme Salma, Min Xue, Md Sayed Ali Sheikh, Xiaoming Guan, Bin Xu, Aiqian Zhang, Lihua Huang, Dabao Xu

**Affiliations:** ^1^Department of Gynecology, Third Xiangya Hospital of Central South University, 138 Tongzipo Road, Changsha, Hunan 410013, China; ^2^Department of Cardiology, Xiangya Hospital, Central South University, Changsha, Hunan, China; ^3^Department of OB/GYN, Baylor College of Medicine, 6651 Main Street, 10th Floor, Houston, TX 77030, USA; ^4^Center for Medical Experiments, Third Xiangya Hospital, Central South University, Changsha, Hunan, China

## Abstract

The aim of the study was to evaluate the role of Smad3, Smad7, and TGF-*β*1 in intrauterine adhesion (IUA) patients and experimental rabbit models. 60 IUA patients, 30 control participants, and 18 female rabbits were enrolled in this study. We found that the plasma concentrations and protein expressions of TGF-*β*1 were significantly increased in patients and experimental rabbits compared to those in controls (*P* < 0.05). Furthermore, the mRNA and protein expression levels of Smad3 were significantly elevated, while Smad7 level was markedly decreased in the patients and experimental rabbits compared with controls (*P* < 0.05). This altered ratio recommended that IUA was positively correlated to the mRNA and protein expression levels of Smad3, Smad7, and TGF-*β*1 in blood and uterine tissue. Moreover, we used the specific inhibitor of Smad3 (SIS3) in experimental rabbit. SIS3 obviously reduced the mRNA and protein expression of smad3 and TGF-*β*1, while it increased Smad7 expression in the treatment groups as compared with IUA rabbits (*P* < 0.05). Our study suggested that TGF-*β*1/Smad3/smad7 is a major pathway which plays an important role in the regulation of the IUA and specific inhibitor of Smad3 (SIS3) may provide a new therapeutic strategy for IUA.

## 1. Introduction

Intrauterine adhesion (IUA) is one of the important causes for infertility or recurrent pregnancy loss. In the past few years, IUAs represented the significant clinical problem of women who mostly oppose infertility and there is no ideal therapy and thus it represents a major unmet medical need. Intrauterine adhesion results from trauma to increase the endometrial fibrosis. On the other hand IUAs are bands of scar-link tissue that form between two surfaces inside the uterine cavity [1]. It has been more than a century since Heinrich Fritsch [2] first described a case of posttraumatic intrauterine adhesion. In general, adhesions are composed of fibrotic tissue, which may result in the adherence of opposing surfaces.

It has been identified that the tissue injury is the specific risk factor and it is possible that, after an injury to the endometrium, fibrosis may follow with the potential for adhesion formation [3]. Conversely, in the tissue the fibrin is rapidly deposited after trauma; then it either reverts or turns into fibrous adhesions [4]. It varies still questionably in patients with experience of resorption or adhesion formation after trauma. In addition the characterizations of tissue fibrosis are considered to arise due to a failure of the normal wound healing response [3, 5]. During normal wound healing, a network of negative feedback mechanisms activated after successful healing is responsible for the proper termination of the proliferative and fibrotic processes, thus restoring tissue integrity. Deregulation of this healing process results in subsequent continuous secretion and deposition of ECM that may lead to the development of tissue fibrosis. In contrast, the repetitive trauma to the tissue may lead to continuous activation of fibrin proliferative response. However, the exact cause of deregulation healing process and continuous deposition of ECM components is still enigmatic. Conversely, the facts of clinical experience of fibrosis may consider as a somewhat permanent scar tissue, during which resolution of the healing process does not occur. Fibrosis is still a major clinical problem, the region of insufficient pathological understanding. TGF-*β*1 stimulates synthesis of ECM proteins and inhibits matrix degradation, resulting in the promotion of fibrosis and tissue repair. TGF-*β*1 belongs to a family of growth factors of critical importance in wound healing and fibrogenesis. Also, platelets are an abundant source of TGF-*β*1; this isoform is released by degranulating platelets at the site of an injury, hence TGF-*β*1 isoform consideration to play the essential role in wound healing and possible subsequent fibrosis [6].

It has been studied extensively in models of liver, kidney, and lung fibrosis [7–9]. The TGF-*β*1 is secreted as part of an inactive tripartite complex consisting of a homodimer of the TGF-*β*1 with latency associated peptide (LAP) and a molecule of latent TGF binding protein (LTBP) [10]. The complex is transported to the ECM and is the major form of TGF*β*1 found in vivo [10, 11]. LTBPs interact with various matrix components including collagen and fibronectin [12]. Nevertheless, TGF-*β*1 is an important growth regulator of human endometrium; the various cellular responses elicited by TGF-*β*1 are mediated through the activation of serine/threonine kinase receptors from the cell surface to the nucleus. Once activated, TGF-*β*1 binds its receptors and then activates its downstream signaling mediators of Smad3, to exert its biological activities [13–16]. Altered expression of TGF-*β*1 in human endometrium has also been associated with various abnormalities including extracellular matrix deposition and adhesion formation [17–19]. Deregulation of the TGF-*β*1/Smads signaling pathway is responsible for tissue fibrosis in several organs. On the other hand, the involvement of Smad3 is observed in fibrosis in many animal models of scleroderma, cystic fibrosis [20, 21]. Verrecchia et al. [22] identified a number of collagen gene promoters in human dermal fibroblasts which were induced by TGF-*β*1 and it depends upon Smad3. It has been recognized that the several abnormalities of endometrium occur with the action of TGF-*β*1 which Smads mediate [23, 24].

It is well accepted that Smad3 is a critical downstream mediator responsible for the biological effects of TGF-*β*1. Alternatively, Smad7 is an inhibitory Smad and negatively regulates Smad3 activation by its negative feedback mechanism. Expression of Smad7 is induced by TGF-*β*1, which, in turn, exerts its negative feedback mechanism by causing degradation of T*β*RI and Smads [25]; however, the overt expression of TGF-*β*1 and Smad3 and decreased Smad7 are mostly observed in renal [26], cardiac [27], and bleomycin (BMI) induced lung fibrosis [28]; this above evidence suggests that TGF-*β*1 acts by stimulating Smad3 to mediate fibrosis.

Conversely, given the importance of TGF-*β*1 in regulating several endometrial cellular activities, the expression and regulation of Smads are necessary to mediate these actions. Besides, specific inhibitor of Smad3 (SIS3) to inhibit endothelial-myofibroblast transition and renal fibrosis in a type 1 diabetic kidney disease demonstrates a therapeutic potential for kidney disease by targeting Smad3 signaling [29]. On the other hand, SIS3 is a specific inhibitor of Smad3 phosphorylation via inhibiting the effects of TGF-*β*1. Furthermore, it has been reported that SIS3 abolishes the ECM overexpression in the normal dermal fibroblasts and scleroderma fibroblasts in vitro [30].

However, the relationship between IUA and Smad3, Smad7 with TGF-*β*1 remains unknown.

Based on the knowledge of the basic properties of TGF-*β*1, we aimed to investigate TGF-*β*1/Smad3 signaling pathway and regulatory mechanisms of inhibitory Smad7 may be essential in IUA remodeling. Therefore, we demonstrated the expression of TGF-*β*1, Smad3, and Smad7 in both IUA patients and experimental rabbit model. Furthermore, we designed a study to evaluate the possible therapeutic effects of SIS3 in experimental rabbit model.

## 2. Materials and Methods

**Figure 1:**
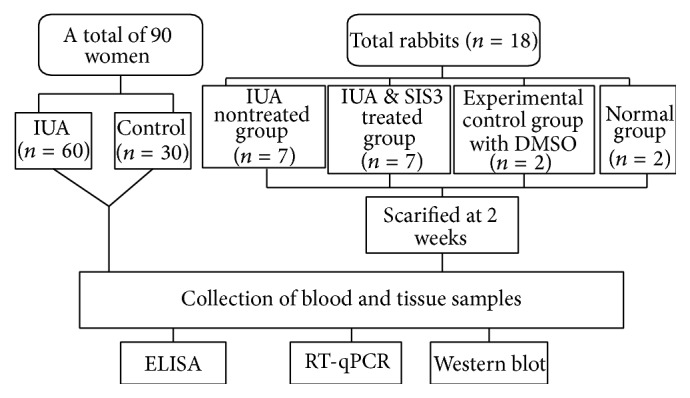
Overview of study design.

The overview of study design is represented in Figure 1.

### 2.1. Human Experimental Protocol

#### 2.1.1. Study Groups

We carried out our study on 60 patients of IUA, mean age 28.06 years (range 19–34), taking a sample of peripheral venous blood and eleven samples of endometrial fibrosis tissue from March 2014 to December 2014, that were enrolled in this study and that were selected to be of moderate type of the IUA. The score and the grade of the IUA were evaluated during hysteroscopy examination according to the revised criteria (Table 1) of the American Fertility Society (AFS), finding where tissue samples from patients undergoing hysteroscopy adhesiolysis for infertility are. Exclusion criteria were infection, any disease in the uterus, chronic inflammatory and malignant disease, and any major operation within the previous 3 months. All patients had signed a written informed consent before surgery and had agreed to the collection of tissues for research.

#### 2.1.2. Control Groups

We carried out our study on 30 fertile women with mean age 28.43 years (range 19–34) taking samples of peripheral venous blood and endometrial tissue biopsy of nonpathological site of uterine cavity to observe the expression of TGF-*β*1, Smad3, and Smad7; inclusion criteria of control women included normal menstrual cycles, no history of previous reproductive surgery or chronic systemic disease, and no hormonal or immune modulator therapy for at least 6 months before the initial examination and being matched for age, having hormone reports within normal limit, and not being hospitalized for at least 1 month prior to participation. The study was approved by the Local Medical Ethics Committee of the Third Xiangya Hospital, Central South University, Hunan, China. All the participants provided written informed consent at the time of enrollment.

### 2.2. Rabbit Experimental Protocol

Experimental protocols were approved by the Institutional Animal Ethics Committee of the Third Xiangya Hospital, Central South University, Hunan, China. From July 2, 2014, to July 23, 2014, 18 mature female fertile New Zealand white rabbits weighing 2500–3000 g, age 5–8 months, were enrolled in this study. They had free access to food and water under standardized laboratory conditions in a temperature and light controlled environment for 2 weeks.

### 2.3. IUA Rabbit Models

Surgeries in rabbits were performed by the same operator. Female rabbits weighing 2500 to 3000 g (age 5-6 months) were used in all experiments, and each underwent operation under aseptic conditions after induction of general anesthesia with pentobarbital sodium (30 mg/kg). Our aim was to propose a model of the pathogenesis condition in the rabbit similar to intrauterine adhesion observed in the human. Rabbits were killed with an overdose of pentothal 2 weeks after the operation. Tissues were cut out and collection of inferior vena blood of rabbits 2 weeks after operation was performed. Fourteen rabbits submitted to experimental curettage. Totally eighteen female rabbits (Table 2) were randomly divided into four groups.

#### 2.3.1. Intrauterine Adhesion Nontreated Group

Seven rabbits were operated for a 0.5–1.0 cm longitudinal incision in the middle of the uterus, and the upper or lower half of the endometrium was randomly scraped using the sharp side of a uterine curette. All rabbits were sacrificed after operation each at 2 weeks after curettage and the abdominal incisions of the rabbits were sutured after flushing the uterine and peritoneal cavities. This method resulted in the development of adhesions within 2 to 3 weeks. Tissues were cut out and collection of inferior vena blood of rabbits 2 weeks after operation was performed.

#### 2.3.2. Intrauterine Adhesion and SIS3 Treated Group

After uterine curettage, performed in an identical manner to that of the endometrial curettage group, then 3 *µ*M SIS3 (SIS3 was used after diluting with solution in DMSO) was intrauterine applied; the abdominal incisions of the rabbits were sutured in 2 layers with continuous 2-0 chromic catgut, after flushing the uterine and peritoneal cavities. Rabbits were killed for the collection of uterine tissue and inferior vena blood 2 weeks after operation.

#### 2.3.3. Experimental Control Group

Two rabbits were operated and DMSO solution in the uterus was injected for 2 weeks as an experimental control group. Tissues were cut out and collection of inferior vena blood 2 weeks after operation was performed.

#### 2.3.4. Normal Group

**Figure 2:**
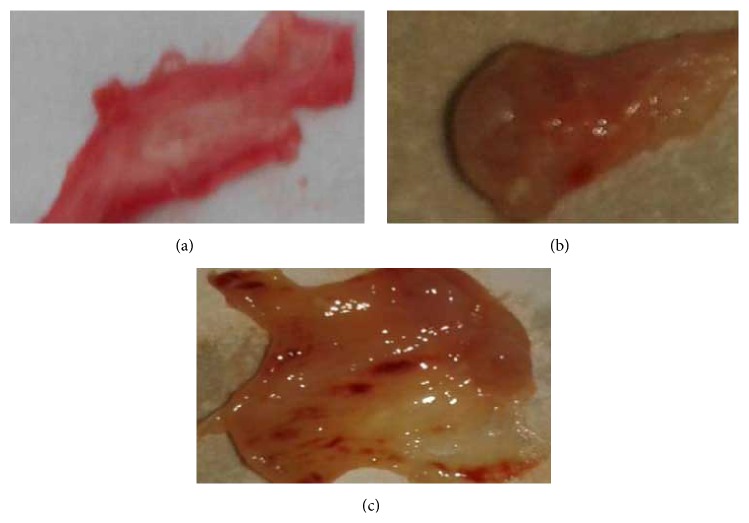
Macroscopic appearance of rabbit uterus. (a) Cut section shown normal uterine endometrium. (b) The IUA rabbit established by endometrial curettage, after 2 weeks, and shown adherence of uterine wall. (c) Following SIS3 intrauterine administration only slight damage endometrial.

In the normal group (*n* = 2), were euthanized only for collection of endometrial tissue and blood samples which were obtained from the inferior vena cava. The macroscopic appearance of the rabbit uterus is represented in Figure 2.

### 2.4. Collection of Blood and Tissue Samples from Patients and Rabbits

Peripheral 5 mL blood samples (human and rabbit) were obtained in K2-EDTA coated tubes and processed within 30 min of collection using two-step centrifugation. Samples were first centrifuged at 1.500 ×g for 15′ at 4°C. The supernatant was collected and then centrifuged again at 14.000 ×g for 15′ at 4°C to obtain pure plasma. Finally, plasma was transferred to RNase-free tubes and stored at −80°C until RNA extraction. Furthermore, tissues of both IUAs and controls (human and animal) were snap frozen in liquid nitrogen and then stored at −80°C until use.

### 2.5. Measurement of TGF-*β*1

We measured transforming growth factor-*β*1 (TGF-*β*1) in plasma obtained from human and rabbit groups by an enzyme-linked immunosorbent assay (ELISA) according to manufacturer's instructions.

### 2.6. Analysis of mRNA of Smad3 and Smad7 by RT-qPCR

We used real-time quantitative reverse-transcription polymerase chain reaction (RT-qPCR) to detect the mRNA expression of Smad3 and smad7 in human and rabbit tissue. Total RNA from different samples was isolated by using TRIzol reagent (TaKaRa, Dalian, China) and the concentration and purity of RNA were determined spectrophotometrically. Firstly, 4 *μ*L of pure RNA (OD 1.8–2.2) was reverse-transcribed (RT) to cDNA at 42°C for 30 minutes using reverse-transcription kits (TaKaRa, Dalian, China) according to the instructions of the manufacturer, through an RT-PCR System (Bio-Rad, USA). Secondly, 2 *μ*L of cDNA was used as the template in qRT-PCR. Then, Smad3 and smad7 mRNA expression were detected by using SYBR Premix Ex Taq (TaKaRa, Dalian, China) according to the manufacturer's protocol, using a 7300 Real-Time PCR System (Applied Biosystems, CA, USA). Briefly, a 10 *µ*L reaction mixture containing 2 *µ*L cDNA template, 5 *µ*L SYBR Master Mix, 0.20 *µ*L ROX, 2.4 *µ*L H_2_O, and 0.20 *µ*L of each primer was amplified by the following thermal parameters: initial incubation at 95°C for 15 s, followed by 40 cycles of denaturation at 95°C for 5 s, annealing, and extension at 60°C for 31 s. The real-time PCR primers were shown in Table 3. Data analysis was performed by comparative Ct method using the ABI software. For the measurement of Smad3 and Smad7 mRNA expression, *β*-actin was served endogenous control and the results were expressed as the ratio of Smad3, Smad7 mRNA to *β*-actin mRNA.

### 2.7. Western Blot Analysis

Human and rabbit tissues were harvested and lysed with ice-cold protein lysis solution and protein lysates were centrifuged at 12,000 g for 15 min at 4°C. Total protein in the supernatant was determined by a BCA Protein Assay Kit (Beyotime, Shanghai, China). Samples containing 30–60 *μ*g of proteins were separated by 10% SDS-PAGE gel and transferred to polyvinylidene fluoride (PVDF) membranes. The membranes were blocked with 5% nonfat milk in TBST buffer (100 mM NaCl, 10 mM tris-HCl, pH 7.4, and 0.1% Tween-20) for 1 hr at room temperature. The membranes were then incubated with primary antibodies against Smad3, P-Smad3 (1 : 200; Santa Cruz, CA, USA), Smad7 (1 : 500; Santa Cruz, CA, USA), TGF*β*1 (1 : 200; Santa Cruz, CA, USA), or *β*-actin (1 : 4000; Santa Cruz, CA, USA) with gentle agitation at 4°C for 18 h, followed by horseradish peroxidase-conjugated secondary antibodies (goat anti-rabbit IgG, 1 : 1000; Santa Cruz; anti-mouse IgG, 1 : 6000; Santa Cruz) for 60 min at room temperature.

The protein bands were determined by Luminata Crescendo Western HRP Substrate (Millipore, Billerica, MA, USA) through Molecular Imager ChemiDoc XRS System (Bio-Rad, Philadelphia, PA, USA). Quantity One 1-D Analysis Software (National Institutes of Health, USA) was utilized for densitometry analysis. Finally, the protein expression results were measured as the ratio of target (Smad3, P-Smad3, Smad7, or TGF*β*1) density to endogenous control (*β*-actin) density.

### 2.8. Statistical Analyses

Data were analyzed with SPSS software (version 20.0, SPSS, Chicago, IL) and reported as mean ± standard deviation (SD). Differences among groups were compared using Student's *t*-test, one-way ANOVA for categorical variables, Fischer's exact test, or the chi-square (*χ* ^2^) test and for non-Gaussian distribution, rank transformation and Wilcoxon test were used. The Smads and TGF-*β*1 expression data were used and graphs were made with GraphPad Prism version 6 for Windows (GraphPad Software, San Diego, CA, USA) and presented as mean ± standard error. All tests were 2-sided and a significant leveled *P* < 0.05 was considered to be statically significant.

## 3. Results

### 3.1. Clinical Characteristics of the Study Groups

A total 60 IUA patients and 30 control women matched for age, history of normal menses, hypomenorrhea, cyclical lower abdominal pain, and abortion were enrolled in this study. The laboratory findings including RBC, WBC, Hb, FSH, LH, prolactin, estrogen, progesterone, and testosterone hormones records were collected, respectively. There were no statistical differences between study group and control group (*P* > 0.05). The details, clinical characteristics of the study group were shown in Table 4.

### 3.2. Plasma Concentration of TGF-*β*1 in IUA Patient and Experimental Rabbit Model

**Figure 3:**
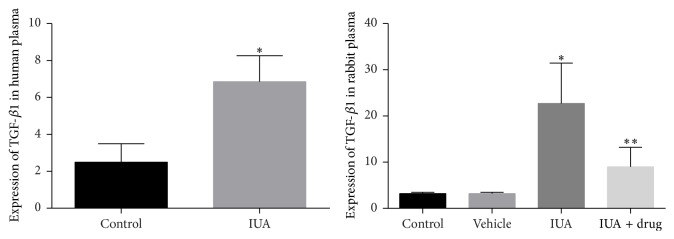
Plasma concentration of TGF-*β*1 level was increased in IUA patients and rabbit compared to that in controls. After administration of SIS3, a significantly decreased plasma TGF-*β*1 level in the treated groups compared with nontreated groups of IUA rabbit. All data were expressed as the means ± SEM. ^*∗*^ *P* < 0.05 versus controls, ^*∗∗*^ *P* < 0.05 versus IUA.

We measured plasma concentration of TGF-*β*1 in IUA patients and intrauterine adhesion nontreated group of rabbit to determine whether TGF-*β*1 was correlated with IUA or not (Figure 3). We found TGF-*β*1 was significantly increased 2.7- and 7.1-fold in IUA patients and intrauterine adhesion nontreated group of rabbit, compared to that in controls (*P* < 0.05). Thus, increased concentration of TGF-*β*1 is established as a key factor in IUA and Smad3 is proposed as a major player in TGF-*β*1 signaling pathways that lead to IUA. In this conception, further study to demonstrate the therapeutic effect of SIS3 in the expression of TGF-*β*1 in intrauterine adhesion and SIS3 treated group of rabbit was performed and it was examined by using ELISA. We found a marked 2.5-fold decrease of TGF-*β*1 in intrauterine adhesion and SIS3 treated group compared to intrauterine adhesion nontreated group of rabbit (*P* < 0.05).

Our results indicate SIS3 exhibited a strong inhibitory effect of TGF-*β*1 signaling in IUA via the inhibition of smad3. Also, Smad3 is considered to be the primary signaling molecule involved in TGF-*β*1 signal transduction in IUA, because TGF-*β*1 elicits potent profibrotic responses in IUA and Smad3, which are activated by TGF-*β*1 and are indispensable for most of its profibrotic actions. Therefore, the inhibition of Smad3 is a necessary component for repressing IUA, which is inhibited TGF-*β*1 that enhances reduction of the occurrence of the IUA. Furthermore, we confirmed this result within tissue fibrosis in IUA patients and experimental rabbit by RT-qPCR and Western blot analysis.

### 3.3. Tissue Expression of TGF-*β*1, Smad3, and Smad7 in IUA Patient and Experimental Rabbit Model

**Figure 4:**
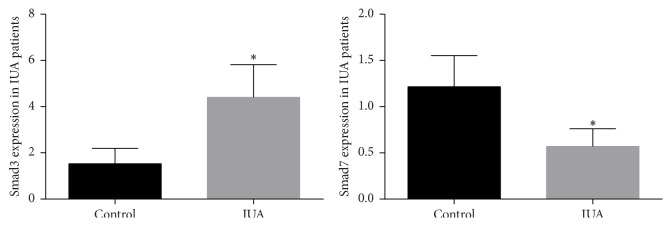
Tissue mRNA expression of Smad3 was increased while Smad7 was decreased in IUA patients compared with controls. All data were expressed as the means ± SEM. ^*∗*^ *P* < 0.05 versus control.

It has been evident that TGF-*β*1 plays an essential role in fibrosis, which is regulated by Smad3. It is well known that the TGF-*β*1/Smad3 signaling system for an autoinhibitory loop involves Smad7; as a result, the expression of Smad7 influences the activity of TGF-*β*1. As an initial experiment, we measured the fibrous tissue expression of mRNA and protein Smad3, Smad7, and TGF-*β*1 in patients and experimental induced rabbits to determine whether TGF-*β*1/Smads differentially regulate their expression in IUA or not. Consistent with the RT-qPCR (cDNA marker), revealed that mRNA expression of Smad3 was significantly increased 2.8-fold, while mRNA expression of Smad7 was significantly decreased 2.1-fold in patients compared with controls (*P* < 0.05) (Figure 4).

**Figure 5:**
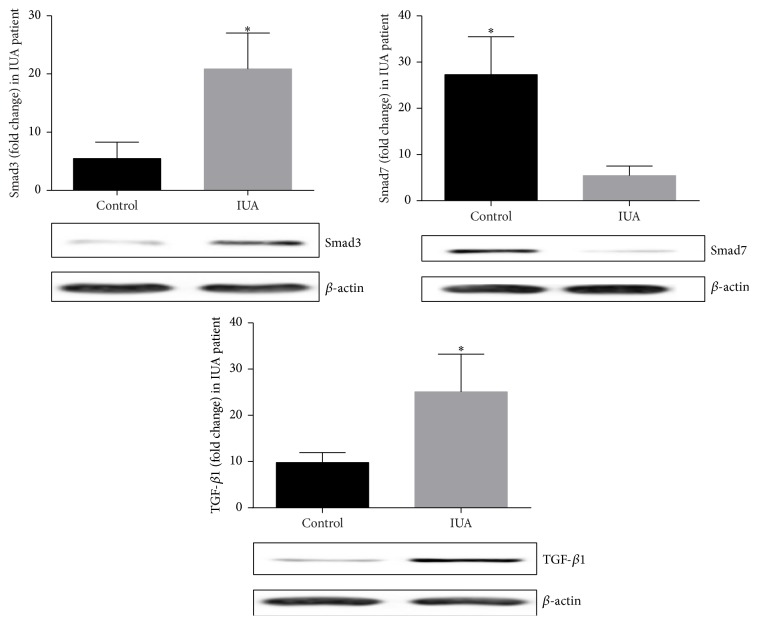
Tissue protein expressions of Smad3 and TGF-*β*1 were increased, while Smad7 was decreased in IUA patients compared with controls. *β* actin for loading controls. All data were expressed as the means ± SEM. ^*∗*^ *P* < 0.05 versus control.

Further, our study demonstrated protein expression of TGF-*β*1, Smad3, and Smad7 by Western blot. Significant 3.8- and 2.5-fold increases of protein expression of TGF-*β*1 and Smad3 were noticed while protein expression of Smad7 significantly decreased 5.01-fold in patients compared to controls (*P* < 0.05) (Figure 5).

**Figure 6:**
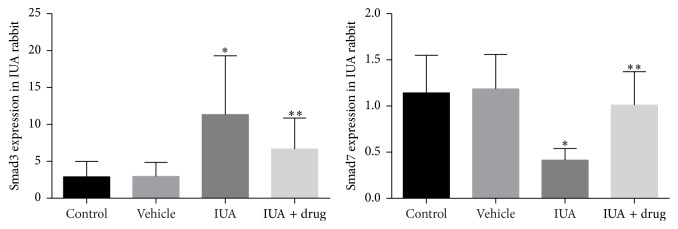
Tissue mRNA expression of Smad3 was increased, while Smad7 was decreased in IUA rabbit compared with controls. After use of SIS3, the mRNA expression of Smad3 was decreased, whereas Smad7 was increased in the treatment groups compared with the nontreated group of IUA rabbits. All data were expressed as the means ± SEM. ^*∗*^ *P* < 0.05 versus control, ^*∗∗*^ *P* < 0.05 versus IUA.

**Figure 7:**
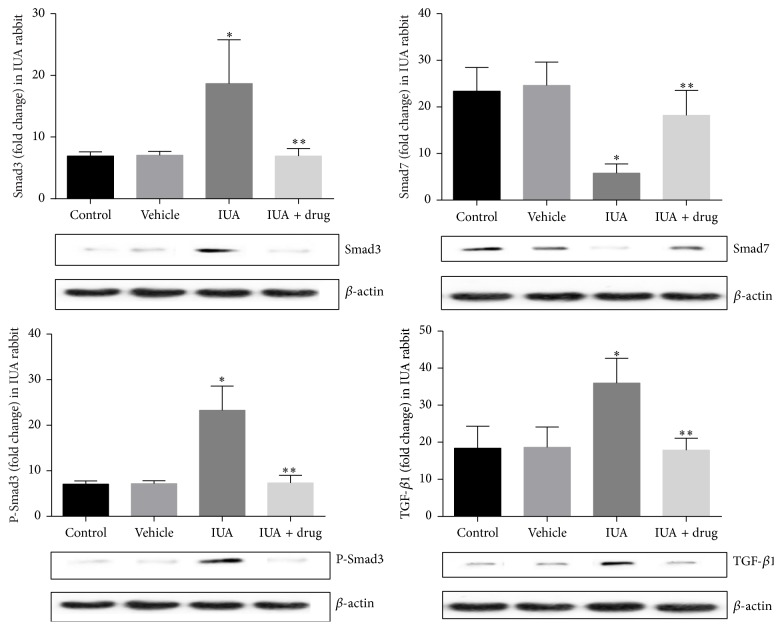
Tissue protein expressions of Smad3, phosphorylation (P) Smad3, and TGF-*β*1 were increased, while Smad7 was decreased in IUA rabbit compared with controls. DMSO was used as a vehicle control. After use of SIS3 the protein expressions of Smad3, P-Smad3, and TGF-*β*1 were decreased, whereas Smad7 was increased in the treated group compared with the nontreated group of IUA rabbit; *β* actin for loading controls. All data were expressed as the means ± SEM. ^*∗*^ *P* < 0.05 versus control, ^*∗∗*^ *P* < 0.05 versus IUA.

The mRNA expression of Smad3 was significantly increased 3.9-fold, while mRNA expression of Smad7 was significantly decreased 2.7-fold in experimental IUA rabbit compared with controls (*P* < 0.05) (Figure 6). In this conception, we therefore examined the protein expression of p-Smad3, Smad3, Smad7, and TGF-*β*1, in IUA rabbit (Figure 7). Total protein expressions of Smad3, P-Smad3, and TGF-*β*1 were markedly increased 2.6-, 3.2-, and 2.01-fold while protein expression of Smad7 was significantly decreased 4.04-fold compared to that in controls (*P* < 0.05).

Similar results were obtained using fibrous tissue of experimental rabbit, in contrast to IUA patient. However, these findings suggest that increased Smad3 and decreased Smad7 are an important mechanism underlying the action of TGF-*β*1 activity in IUA.

### 3.4. Effect of SIS3 on Smad3, P-Smad3, Smad7, and TGF*β*1 in Experimental Rabbit Model

Next, we investigated the possible effects of specific inhibitor of Smad3 (SIS3 with dose of 3 *µ*M) in IUA which was associated with increased Smad3, P-Smad3, and TGF*β*1 and decreased Smad7. It has been reported that SIS3 with a dose of 3 *µ*M is able to reduce the Smad3 and inhibited Smad3 phosphorylation and TGF-*β*1 induced fibrotic response in fibroblasts [30]. Few studies have demonstrated that pretreatment of cells with SIS3 (3 *µ*M) in human dermal fibroblasts was completely abrogated to TGF-*β*1 signaling that contributes to the upregulation of type I procollagen via Smad3 [30]. Consistent with these findings, the present study demonstrates that the expression of fibrous tissue mRNA Smad3, Smad7 and protein of Smad3, P-Smad3, Smad7, and TGF-*β*1 were determined by RT-qPCR and Western blot analysis, respectively (Figures 6 and 7). We found the mRNA and protein expression of Smad3 were markedly decreased 1.6- and 2.6-fold, while Smad7 was significantly increased 2.4- and 3.1-fold in the intrauterine adhesion and SIS3 treated group compared to intrauterine adhesion nontreated group (*P* < 0.05). We also found the P-Smad3 was decreased 3.1-fold in treated group compared to nontreated group of IUA rabbit (*P* < 0.05). Further, we found fibrous tissue protein expression of TGF-*β*1 was significantly decreased 2.01-fold in the intrauterine adhesion and SIS3 treated group compared to the intrauterine adhesion nontreated group (*P* < 0.05).

Together, these results indicated that SIS3 inhibits the upregulation of Smad3 by the suppression of the Smad3 phosphorylation and increased Smad7 was associated with inhibitory effect of TGF-*β*1 in IUA. However SIS3, which inhibited increased TGF-*β*1 signaling, could be used as a therapeutic intervention for IUA via the inhibition of Smad3.

## 4. Discussion

This study described for the first time the role of individual expression of TGF-*β*1, Smad3, and Smad7 signal pathway in IUA patients and experimental rabbit model. We investigated in patients and created a rabbit model of postoperative IUA using the endometrial curettage to establish a similar condition of IUA in human, which will be help in our understanding of molecular mechanisms of intrauterine adhesion and also help to evaluate appropriate therapeutic alternative for prevention and treatment of the IUA. In the past decades, extensive studies have been recognized in the role of various cytokines and growth factors that can lead to the development of tissue fibrosis in different organs. Among them, TGF-*β*1 is a multifunctional regulatory cytokine with crucial roles in fibrotic processes. Therefore, it is important that only a few studies about the potential role of TGF-*β*1 in fibrosis have been published so far [31, 32]. It is well accepted that active TGF-*β*1 binds its receptors and then activates its downstream mediators, Smad2 and Smad3, to exert its biological effects, including fibrosis [33, 34]. The basis for TGF-*β*1 regulation of collagen synthesis has developed rapidly as previous studies identified by Smad family [35]. However, Smads component is a major specific signal transduction pathway of TGF*β*1 [35]. In the present study, we determine the expression of TGF-*β*1, Smad3, and Smad7 levels in IUA patients and experimental rabbit model to identify the molecular defects underlying the TGF-*β*1 action. We divided three experimental approaches: in the first approach we demonstrated patient plasma for TGF-*β*1 and fibrous tissue for mRNA and protein that has expressed the regulatory Smad3 and inhibitory Smad7 in addition to TGF-*β*1, respectively.

In the second approach, we establish a rabbit model of IUA using the endometrial curettage. The delay in the regeneration of endometrial epithelial cells may be important to the formation of IUA after endometrial damage. Because of the insufficient epithelial cells covering the uterine cavity, the stroma is bared, allowing fibroblast activity and connective tissue formation leading to endometrial fibrosis and possibly to the development of the IUA. With our experimental design the whole process of injury or trauma caused by sharp, deeply endometrial curettage could be evaluated and the partial clearance of endometrium was preceded that may contact the bare endometrium or myometrium and then caused more injury effect. Due to epithelial loss or epithelialization delay, the presence of fibrosis, or the varying degrees of scars, adhesions began to appear. Usually the epithelialization did not start until 2 weeks after injury; considerable granulation tissue, hyperplasia, and fibrosis appeared. When a sufficient percentage of fibrosis appears, it will hinder the regeneration of the endometrium and formation of the adhesion [36, 37]. However, trauma to endometrium goes through pathological changes and limited repair consistent with the clinical observation in IUA patient.

Our purpose was a model of the pathogenesis condition in the rabbit similar to IUA observed in the patient. Indeed, our initial experiment of rabbits, the time points of 2 weeks after endometrial curettage has been characterized as the early stage of IUA with healing scar may produce fibrosis overt an IUA stage respectively, despite the fact that TGF-*β*1 has powerful stimulation in tissue adhesions and continuous TGF-*β*1 activation is associated with pathological cause of the IUA.

In the present study, confirms to establish IUA a rabbit model by visible to the naked eye, plasma concentration of TGF-*β*1 and tissue mRNA and protein expression of Smad3, Smad7, and TGF-*β*1.

In the third approach, the study was to assess the possible therapeutic effect as a treatment of intrauterine application of SIS3 after endometrial curettage of IUA rabbit that was compared with the intrauterine adhesion nontreated group of rabbit.

ELISA analysis was used to determine the expressions of plasma TGF-*β*1, RT-qPCR were used to determine Smad3 and Smad7 mRNA, and Western analysis was used to determine total Smad3, P-Smad3, Smad7, and TGF-*β*1 protein expression in both patient and rabbit model. In the present study, we demonstrated that the plasma concentration and protein expression of TGF-*β*1 levels significantly increase in IUA patients and experimental rabbit model compared to those in controls, respectively (*P* < 0.05). Previously, data showed that increased TGF-*β*1 induces fibroblasts to synthesize and contract ECM; this cytokine has long been believed to be a central mediator of the fibrotic response [10, 27].

On the basis of this conception our results indicate increased expression of TGF-*β*1 levels have preceded the formation of fibrosis that enhances the development of the IUA. Consistent with our observation, increased TGF-*β*1 has been shown in atrial fibrosis, as well as transient overexpression of porcine TGF-*β*1 in rat lungs using the adenovirus transfection system, induced prolonged lung fibrosis. Moreover, TGF-*β*1 is expressed at greater levels in keloid fibroblasts when compared with normal dermal fibroblasts. There was an also growing evidence support that TGF-*β*1 stimulates the progression of cardiac fibrosis during cardiac hypertrophy and heart failure [38].

Indeed, in the present study, we found the marked different expression of tissue mRNA and protein smad3, Smad7 in patients and experimental rabbit model; we addressed this issue and found in other fibrotic disease; therefore our study believes these different expressions of Smad may alter the development of the IUA.

However, the expression of Smads in IUA as well as their isolated adhesions tissue indicates that these adhesions possess the necessary components of the TGF-*β*1 signaling pathway that can be recruited and activated by Smads component. A significantly increased TGF-*β*1 level had an effect on the expression of Smad3 and Smad7 in the IUA. It has been evident that Smad3 plays a role in the development of pathological fibrosis in different organs; for consistence in this regard, we further demonstrate the tissue mRNA and protein expression of Smad3 levels in IUA.

We found the tissue mRNA and protein expression of Smad3 levels are significantly increased in patients and experimental rabbit model compared to those in controls (*P* < 0.05). We also measured phosphorylation of Smad3 that significantly increased in the experimental IUA rabbit model compared to controls (*P* < 0.05).

Our result suggests the altered TGF-*β*1 expression has changed the Smad3 expression and activation that may be more important in inducing the fibrosis in the IUA. However, increased expression of Smad3 is equally important as an increased expression of TGF-*β*1 in the development of the IUA. Also, TGF-*β*1 is acting as a key regulator to change the Smad3 expression in IUA. Thus, previously published data taken together with our present study propose the existence of species specificity in the profibrotic effects of TGF-*β*1 on endometrial stroma [17, 25–28]. Consistent with our observation, few studies have shown increased Smad3 expression in scleroderma skin, scleroderma fibroblasts, and postinfarction myocardial fibrosis [27, 28, 39]. Takagawa et al. [40] also demonstrated the expression of Smad3 increased in a murine model of BM-induced scleroderma; these observations have initiated and facilitate our study to establish that Smad3 is an important step in signal transduction or the activation of the IUA.

In the present study, we believed the IUAs occur due to the abnormal responses or functional activities exhibited by tissue fibroblasts towards the mediators of TGF-*β*1 which Smads mediate. On the other hand, few studies demonstrated the evidence that found an underlying correlation between decreased Smad7 and skin lesions, in a murine model of scleroderma and myocardial fibrosis in infracted heart [20, 21, 28]. It was previously demonstrated that the Smad7 present within the cytoplasm of endometrium that regulates TGF-*β*1 ligand initiated signaling by competing with the receptor regulated Smads for receptor based phosphorylation and activation by an autocrine/paracrine action of TGF-*β* [7]. Generally Smad7 not only is constitutively expressed, but also appears to be rapidly induced by TGF-*β*1 in different fibroblast including dermal primary fibroblasts [20, 21].

On the basis of our data increased levels of Smad7 are opposed to the fibrotic actions of TGF-*β*1 in IUA, whereas decreased levels of Smad7 are more responsible for fibrotic effect in IUA. Consistent with these findings, our result indicates the IUA associated with marked decrease of tissue mRNA and protein expression of Smad7 and it will probably challenge the homeostatic role of Smad7 in modulating of TGF-*β*1 responses.

As an inhibitory regulator in the TGF-*β*/Smad signaling pathway, Smad7 can be induced by a Smad3-dependent mechanism which in turn blocks the signal transduction of TGF-*β*1 via its negative feedback loop [25, 41–43]. Elevated TGF-*β*1 level and decreased Smad7 level are often present in tissues where an uncontrolled fibrotic response occurs. This finding is consistent with our present study in which we found the tissue mRNA and protein expression of Smad7 levels are significantly decreased in patients and experimental rabbit model compared to those in controls (*P* < 0.05). Decreased Smad7 is induced activation of Smad3 via TGF-*β*1 dependent pathways; as a result Smad3-mediated fibrosis occurs in IUA. Previous studies also reported reducing Smad7 by activating the Smad3-dependent Smurf2 degradation pathway [41, 43]. Reduction of Smad7 is caused by ubiquitin-dependent protein degradation that could be mediated, at least in part, by Smurf2 proteins and that plays an important role in the fibrosis [44]. Consistent with this observation, TGF-*β*1 induces Smurf2 expression via a Smad3-dependent mechanism [44]. However, collectively evidence indicated that dysregulation of Smad3 and Smad7 was established to be one of the major mechanisms in mediating the fibrotic response in IUA. In this regard, dysregulation of Smad3 and Smad7 in IUA may regulate through decreased Smad3 and increased Smad7 simultaneously, which seems to be an effective strategy for treatment of the IUA.

In our experimental rabbit protocol, we observe the expression of Smad3 at 2 weeks overt the control; these observations suggest that the prolonged activation of Smad3 is important for the initiation to activation of TGF-*β*1 signals. However, our experimental rabbit data show that, during the development of IUA, there is an increase in TGF-*β*1 signaling as shown by increase of Smad3 in fibrous tissue; thus this was attempted to activate TGF-*β*1 signaling by fibrous tissue as evidenced by the decreased expression of the Smad7. Therefore, this negative feedback loop appears sufficient for the development of the IUA. The action of TGF-*β*1 is effective in IUA, possibly due to a higher TGF-*β*1 receptor expression in IUA, resulting in increased Smad3 and decreased Smad7 expression.

Further, the present study revealed the therapeutic effects of SIS3 in experimental rabbit model. SIS3 is a potent and selective inhibitor of Smad3 function. TGF-*β*1 is a major stimulus for tissue fibrosis by enhancing the synthesis of collagens and other matrix components [45, 46]. TGF-*β*1 regulation of collagen synthesis has developed rapidly, which Smads mediate [47]. Smad3 are necessary for the transcriptional activation of collagen genes [48, 49]. On the other hand, several studies have evaluated the fibrosis on the basis of the accumulation of collagen in tissue, due to an imbalance between production and deposition of ECM components including collagen type 1, and it promotes increased TGF-*β*1 and Smad3 while decreasing Smad7 [26, 50–52]. TGF-*β*1 signaling contributes to the upregulation of type I procollagen via Smad3 in human dermal fibroblasts [53, 54]. Consistent with the above findings, the present study found overexpression of Smad3 due to increased phosphorylated Smad3 in uterine fibrous tissues. Besides, decreased Smad7 expression resulted in sustained activation of TGF-*β*1 and Smad3 signaling and this trend may be due to a loss of the inhibitory effect of Smad7 on Smad3 activation which is closely associated with fibrosis in IUA. Furthermore, SIS3 has been shown to inhibit collagen synthesis by fibroblasts and fibrosis in several different experimental models. SIS3 may reduce the upregulated expression of type I collagen through the inhibition of phosphorylated Smad3 in scleroderma fibroblasts. From the above evidence, the present study recommended that SIS3 is responsible for inhibition of phosphorylated Smad3 and type I collagen expression in IUA.

Moreover, 3 *µ*M SIS3 completely diminished the constitutive phosphorylation of Smad3 as well as the excessive type I procollagen expression in scleroderma fibroblasts. These findings supported our results that increased phosphorylation of Smad3 is an important cause of excessive ECM deposition and SIS3 is a selective inhibitor of collagen type I that reduced the occurrence of fibrosis in IUA. This finding has showed, consistent with previous studies, treatment with SIS3 in fibrosis of several different experimental models [30]. Therefore, in this study, we had aimed to examine the efficacy of SIS3 in the treatment of an IUA rabbit model. Conversely, we demonstrate the intrauterine application of a specific inhibitor of Smad3 (SIS3) that alters the expression of Smad3 and Smad7 activity, which enhances altering of the transcriptional response of TGF-*β*1 in IUA.

**Figure 8:**
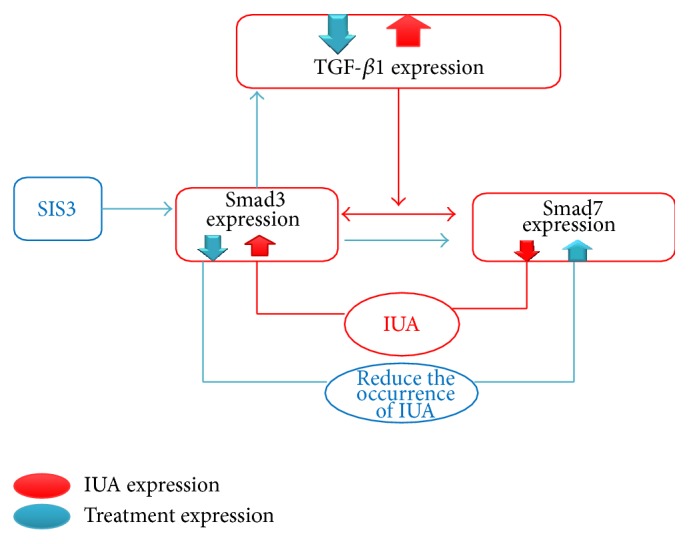
A marked increase of TGF*β*1 and Smad3 but a decrease of Smad7 (red), suggesting that the imbalance between TGF*β*1, Smad3, and Smad7 signaling could be important in the development of the IUA. Inhibitory Smad7 competes with Smad3 for access to the activated TGF-*β*1, thereby preventing Smad3 and blocking TGF-*β*1 induced responses in IUA by SIS3 in the experimentally induced rabbit. We consider it unlikely that increased Smad3 expression plays a major role in altered TGF-*β*1 signaling. However, inhibition of Smad3 and the induction of Smad7 are therefore essential for preventing excessive TGF-*β*1 stimulation (blue) that enhance reduction of the occurrence of the IUA.

In the present study, we found the marked decrease of expression of TGF-*β*1 and Smad3 levels while Smad7 level significantly increased in the intrauterine adhesion and SIS3 treated group compared to intrauterine adhesion nontreated group (*P* < 0.05). Also, SIS3 is a Smad3 inhibitor and blocks Smad3 signaling directly by inhibiting Smad3 phosphorylation. Moreover, SIS3 also acts as a Smad7 agonist and inhibits Smad3 signaling by inducing Smad7, which reduce the fibrosis of IUA (Figure 8). This finding was consistent with other study in which there was inhibition of Smad3 with a combination of naringenin (NG) and asiatic acid (AA) (NG is a Smad3 inhibitor and blocks Smad3 signaling directly by inhibiting Smad3 phosphorylation whereas AA functions as a Smad7 agonist and inhibits Smad3 signaling by inducing Smad7) [55].

However, Smad3 plays such a critical role in mediating the pathobiology of fibrotic disease, as demonstrated by the previously cited studies; inhibition of Smad3 signaling could be a prime target for intervention in fibrotic conditions. Although increased TGF-*β*1 was shown to induce the decreased expression of Smad7, this induction was transient and therefore indicated the existence of a negative feedback loop within the pathway and SIS3 has the ability to alter this pathway rather than enhance reduction of the occurrence of the IUA. In this regard, we are attempting to elucidate the antifibrotic effect of SIS3 in IUA.

Also, SIS3 recognizes the inhibitory receptor of smad3 signal transduction pathways that result in interruption of TGF-*β*1 action. SIS3 acts as an important negative feedback inhibitor of TGF-*β*1 signaling by blocking the activation of Smad3 and preventing their interaction with activated TGF-*β*1 receptors; as a result disruption of Smad3 confers resistance to the development of the IUA.

Together all data in the present study establish that significantly increased the expression of TGF-*β*1, Smad3 while marked decreased Smad7 levels in intrauterine adhesion lead to, alteration of TGF-*β*1 intracellular signaling and the underlying mechanism of TGF-*β*1 action. As a substitute specific inhibitor of smad3 (SIS3) that selectively targets individual signaling pathways downstream of the TGF-*β*1 receptor to significantly decreased TGF-*β*1, Smad3 while increasing Smad7 levels, therefore likely to be more successful to elicit to reduce the fibrosis occurrence in experimental IUA rabbit model. These results suggest that TGF-*β*1 mediates action through Smads, and most likely signaling pathways result in intrauterine adhesion, growth, and its regression by specific inhibitors of Smad3 (SIS3).

## 5. Conclusion

Our findings indicate that alterations in the expression of TGF-*β*1 and Smad3 components occur during the development of IUA in both patient and experimental rabbit model. The patients and rabbit blood have shown significantly increased plasma concentration of TGF-*β*1 is markedly correlated to the development of the IUA, and IUA is promoted to the increased tissue expression of TGF-*β*1 and Smad3 and decreased Smad7. Therefore, treatments targeting the modification of TGF-*β*1, Smad3, and Smad7 signaling via suppressing Smad3 with SIS3 may provide a new therapeutic strategy for IUA.

## Figures and Tables

**Table 1:** The American Fertility Society classification of intrauterine adhesions, 1988.

Extent of cavity involved	<1/3	1/3–2/3	>2/3
	1	2	4
Type of adhesions	Filmy	Filmy & dense	Dense
	1	2	4

Menstrual pattern	Normal	Hypomenorrhea	Amenorrhea
	0	2	4

Stage l (mild) 1–4, Stage ll (moderate) 5–8, and Stage lll (severe) 9–12.

**Table 2:** Rabbit groups with protocol.

Group	Number	Protocol	Scarified
Intrauterine adhesion nontreated group	7	IUA established by endometrial curettage	At 2 weeks
Intrauterine adhesion & SIS3 treated group	7	IUA established by endometrial curettage with applied SIS3 (3 *μ*M) as previously mentioned [30]	At 2 weeks
Experimental control group	2	Rabbits were operated and DMSO solution was injected	At 2 weeks
Normal group	2	Euthanized only for taking samples	At 2 weeks

**Table 3:** Primers used for PCR analysis.

Target mRNA	Primer sequence
Smad3-forward: 5′	CGACCACCAGATGAACCACA-3′
Smad3-reverse: 5′	GCTGTGAAGCGTGGAATGTCT-3′
Smad7-forward: 5′	AACTGCAGACTGTCCAGACG-3′
Smad7-reverse: 5′	GAGGTTGGGAATCTGAAAGC-3′
*β*-actin-forward: 5′	CATCCTGCGTCTGGACCTGG-3′
*β*-actin-reverse: 5′	TAATGTCACGCACGATTTCC

**Table 4:** Clinical and laboratory findings of the study groups.

Variable	IUA (*n* = 60)	Control (*n* = 30)	*P*
*Clinical findings *			
Age	28.06 ± 3.63	28.43 ± 3.44	0.319
Normal menses	—	30	0
Hypomenorrhea	60	—	0
Cyclical lower abdominal pain	24	—	0
Abortion	60	—	0
*Laboratory findings *			
RBC (*∗*10^−^12/L)	4.41 ± 0.28	4.42 ± 0.21	0.948
WBC (*∗*10^−^9/L)	5.38 ± 1.33	5.47 ± 0.83	0.748
Hb (g/L)	131.5 ± 7.79	133.13 ± 6.14	0.089
FSH (mlU/mL)	7.58 ± 1.76	8.18 ± 1.53	0.117
LH (mlU/mL)	7.64 ± 2.44	7.68 ± 1.67	0.209
PRL (ng/mL)	17.59 ± 2.95	16.57 ± 3.86	0.168
Estrogen (pmol/L)	277.25 ± 155.4	282.65 ± 121.91	0.868
Progesterone (0.ng/mL)	0.651 ± 1,02	0.822 ± 0.07	0.366
Testosterone (ng/dL)	28.76 ± 7.61	28.42 ± 5.82	0.831

The data are represented as mean ± SD. FSH = follicle stimulating hormone, LH = luteinizing hormone, and PRL = prolactin. All *P* values are represented comparisons between IUA patient and control groups.
